# Dietary *Magnolia kobus* DC. Attenuates Age-Associated Hippocampal Alterations in Mice in Association with Modulation of Iron Metabolism- and Ferroptosis-Related Markers

**DOI:** 10.4014/jmb.2606.06032

**Published:** 2026-09-15

**Authors:** Jong Min Kim, Hyun-Jin Na, Yiseul Kim, Sang-Hee Lee, Mi Jeong Sung

**Affiliations:** 1 Korea Food Research Institute, Wanju-gun 55365, Republic of Korea; 2 Department of Herbal Crop Research, National Institute of Horticultural and Herbal Science, Rural Development Administration, Eumseong-gun 27709, Republic of Korea; 3 Department of Food Biotechnology, University of Science and Technology, Wanju-gun 55365, Republic of Korea

**Keywords:** *Magnolia kobus* DC., Hippocampal aging, Cognitive dysfunction, Ferroptosis, Iron Homeostasis, Neuroinflammation

## Abstract

Brain aging is a major contributor to cognitive decline and neurodegenerative disorders. Ferroptosis, a regulated form of cell death driven by iron-dependent lipid peroxidation, has emerged as a key mechanism associated with age-related hippocampal dysfunction. Here, we investigated whether dietary supplementation of *Magnolia kobus* DC. (MO) attenuates age-related hippocampal alterations in mice in association with changes in iron metabolism- and ferroptosis-related markers. Behavioral tests combined with molecular and histological analyses of hippocampal tissues from young and aged mice demonstrated that MO significantly attenuated age-associated behavioral alterations, including Y-maze spontaneous alternation performance, and modulated age-related molecular and histological alterations in the hippocampus. MO supplementation was associated with reduced expression of cellular senescence and neuroinflammatory markers and modulation of blood–brain barrier-associated tight junction proteins. Furthermore, MO supplementation was associated with partial normalization of age-associated alterations in iron metabolism-related proteins and modulation of ferroptosis-related markers, including lipid peroxidation-related markers. MO supplementation was also associated with increased cholinergic and synaptic protein expression and lower β-amyloid-related immunoreactivity and p-Tau (Ser404) levels in the aged hippocampus. These findings suggest that dietary MO attenuates age-associated hippocampal dysfunction in association with modulation of iron metabolism- and ferroptosis-related markers, highlighting its potential as a functional dietary intervention for promoting healthy brain aging.

## Introduction

Brain aging is a fundamental biological process associated with progressive cognitive decline and an increased risk of neurodegenerative disorders [1]. Among brain regions, the hippocampus is particularly vulnerable to age-associated structural and functional alterations because of its central role in learning, memory, and synaptic plasticity [2]. Age-associated hippocampal dysfunction is characterized by neuronal and synaptic impairment, disruption of neurotransmitter systems, chronic neuroinflammatory responses, and oxidative stress, all of which contribute to cognitive decline and increased susceptibility to neurodegenerative disorders [3, 4]. Despite extensive research, effective dietary or therapeutic strategies for preventing or delaying hippocampal aging remain limited, highlighting the need to identify novel interventions that preserve hippocampal function during aging [5].

Recent evidence suggests that dysregulation of iron homeostasis and ferroptosis-associated signaling contributes to age-associated cognitive impairment and hippocampal dysfunction [6]. Ferroptosis is a regulated form of cell death characterized by iron-dependent lipid peroxidation and disruption of cellular antioxidant defense systems. It has recently been recognized as a process associated with iron dysregulation and age-related neurodegeneration [7]. Age-associated alterations in iron metabolism may be associated with oxidative stress and lipid peroxidation in the hippocampus, thereby contributing to neuronal dysfunction and synaptic impairment [8]. In the hippocampus, ferroptosis-associated signaling is associated with increased lipid peroxidation, disruption of antioxidant defense systems, and alterations in iron transport and storage proteins, potentially contributing to neuronal loss and cognitive decline [9]. These findings suggest that modulation of iron homeostasis and ferroptosis-associated pathways may represent a potential strategy for attenuating hippocampal aging [10].

*Magnolia kobus* DC. (MO) is a traditional natural material used in East Asian medicine. Previous studies have reported protective effects of MO in experimental models associated with oxidative stress and inflammation [11, 12]. However, its potential role in age-associated hippocampal dysfunction and ferroptosis-associated signaling remains unclear.

In the present study, we aimed to investigate the effects of dietary MO supplementation on hippocampal aging in aged mice, with a focus on iron homeostasis and ferroptosis-associated signaling. Behavioral, molecular, and histological analyses of hippocampal tissue were performed to examine whether dietary MO supplementation was associated with changes in ferroptosis- and iron metabolism-related markers during hippocampal aging.

## Materials and Methods

### Sample Preparation and Characterization

The dried flower buds of *Magnolia kobus* DC. (MO), cultivated in Jeollabuk-do, Republic of Korea, were obtained from a local commercial supplier in Jeonju, Republic of Korea. The plant material was finely pulverized and stored at -20°C until use. For extraction, the powdered MO was mixed with 50 volumes of 50% (v/v) ethanol and incubated at 40°C for 24 h under continuous agitation. The extract was filtered through Whatman No. 2 filter paper (Whatman International Limited, UK), concentrated under reduced pressure using a rotary vacuum evaporator, lyophilized, and stored at −20°C until use. The final extraction yield was 10% (w/w).

The MO extract used in the present study was the same preparation characterized in our previous studies. UPLC-QTOF-MS^E^ analysis tentatively identified nine major components, including three 4-O-(3′-O-D-glucopyranosyl)-caffeoylquinic acid isomers, chlorogenic acid, syringin 4-O-β-glucoside, a patuletin glycoside, magnoloside C, kaempferol 3-(p-coumaroyl-glucoside), and pinellic acid [11]. The total phenolic and total flavonoid contents of the extract were 136.12 ± 6.25 mg gallic acid equivalents/g and 79.31 ± 1.12 mg rutin equivalents/g, respectively [13].

### Animal Experimental Procedures

Male C57BL/6J mice were obtained from Oriental Biotechnology (Republic of Korea) and housed under controlled environmental conditions (22 ± 2°C, relative humidity 50%–60%) with a 12 h light/dark cycle. All animals had ad libitum access to food and water throughout the experimental period. All animal experiments were approved by the Institutional Animal Care and Use Committee of the Korea Food Research Institute (approval number: KFRI-M-24030) and conducted in accordance with institutional ethical guidelines and the ARRIVE guidelines.

Mice were randomly assigned to a young control group (YC, n = 8), an aged control group (OC, n = 8), and an aged MO-supplemented group (OC+MO, n = 8). All eight animals per group were included in the behavioral analyses. Hippocampal samples from six independent animals per group were used for Western blotting. The YC group consisted of 6-month-old mice, while both aged groups consisted of 21-month-old mice at the start of the experiment. The YC and OC groups received the vehicle alone, whereas the OC+MO group received MO at 100 mg/kg/day. The dose of 100 mg/kg/day was selected based on our previous *in vivo* studies in which the same MO extract showed biological efficacy in mouse models of carotid artery ligation-induced vascular remodeling and adenine-induced chronic kidney disease [11, 13]. MO was dissolved in reverse osmosis (RO) water and administered by oral gavage at a dose of 100 mg/kg/day in a volume of 200 μL per mouse. The YC and OC groups received an equivalent volume of RO water (200 μL per mouse) by oral gavage. All treatments were delivered by oral gavage once daily for 12 weeks. Following behavioral assessments, mice were anesthetized with 2.5% isoflurane and euthanized. For molecular analyses, the hippocampus was carefully dissected from fresh brain tissue, snap-frozen in liquid nitrogen, or homogenized in an appropriate lysis buffer for protein extraction.

### Behavioral Tests

Behavioral assessments were performed before tissue collection. Spatial working memory was evaluated using the Y-maze test. The Y-maze consisted of three identical arms, and mice were allowed to freely explore the maze for 8 min starting from a designated arm. Mouse movements were recorded using an automated video-tracking system (Smart 3.0, Panlab, Spain). Spontaneous alternation performance was calculated as the ratio of successive entries into all three arms to the total number of arm entries minus two and was expressed as a percentage. Locomotor activity and anxiety-like behavior were assessed using the open-field test. Mice were individually placed in a square white acrylic arena (50 × 50 × 50 cm) and allowed to explore freely for 5 min. The time spent in the central zone (25 × 25 cm) and the peripheral zone was automatically recorded using the same video-tracking system.

### Immunofluorescence Staining

Paraffin-embedded brain sections were first deparaffinized in xylene and subsequently rehydrated through a descending ethanol series (100%, 95%, 70%, and 50%), followed by rinsing with cold distilled water. Antigen retrieval was performed using a heat-induced epitope retrieval method with an appropriate unmasking solution. After cooling, tissue sections were incubated with 5% bovine serum albumin for 1 h at room temperature to block nonspecific antibody binding. The sections were then incubated overnight at 4°C with primary antibodies against 4-hydroxynonenal (4-HNE; ab46545, Abcam, UK) or prostaglandin-endoperoxide synthase 2 (PTGS2; ab15191, Abcam). Following primary antibody incubation, the sections were washed and incubated with a Cy3-labeled goat secondary antibody (Servicebio, China) for 2 h at room temperature in the dark. Nuclear counterstaining was performed using 4',6-diamidino-2-phenylindole (DAPI). After washing with phosphate-buffered saline containing Tween 20, the sections were mounted with VECTASHIELD mounting medium. Two independent biological samples were analyzed for each group. For each biological sample, fluorescence intensity was measured in at least 10 randomly selected fields of view from each of three to four slides, resulting in the analysis of more than 30 fields of view per group. Fluorescence intensities of 4-HNE and PTGS2 were quantified using Fiji/ImageJ (Olympus FY3000 fluorescence microscope (Olympus Co., Japan)). For each staining experiment, images from all experimental groups were acquired under identical imaging conditions. Regions of interest (ROIs) were defined around individual cells, and the mean fluorescence intensity within each ROI was measured.

### Western Blot Analysis

Proteins extracted from hippocampal tissues were quantified and separated using sodium dodecyl sulfate–polyacrylamide gel electrophoresis, followed by transfer to polyvinylidene fluoride membranes. The membranes were blocked with 3% bovine serum albumin in Tris-buffered saline containing 0.1% Tween-20 at room temperature to minimize nonspecific binding. Subsequently, the membranes were incubated overnight at 4°C with primary antibodies against the indicated proteins. Detailed information on the primary antibodies used in this study is provided in Table S1. After primary antibody incubation, the membranes were washed and incubated with appropriate horseradish peroxidase-conjugated secondary antibodies (Cell Signaling Technology, USA). Protein bands were visualized using a ChemiDoc imaging system (Bio-Rad, USA), and band intensities were quantified using ImageJ software (National Institutes of Health). For each molecular endpoint, Western blot analyses were performed in three independent experimental runs. Each run included hippocampal protein samples from two different animals per group, yielding six independent biological samples per group (n = 6). Band intensities were normalized to β-actin and expressed as relative protein expression. Each animal contributed one independent value to the final statistical analysis.

### Statistical Analysis

All data are presented as the mean ± standard deviation. All statistical analyses were performed using GraphPad Prism software (version 10.0; GraphPad Software, Inc., USA). The normality of each dataset was assessed using the Shapiro–Wilk test, and homogeneity of variance was evaluated using the Brown–Forsythe test. Statistical differences among the three groups were analyzed using one-way analysis of variance (ANOVA) followed by Tukey’s multiple comparisons test. For behavioral analyses, n = 8 represents eight independent animals per group. For Western blot analyses, n = 6 represents six independent biological samples obtained from six individual animals per group and analyzed across three independent experimental runs. Technical replicates or repeated measurements of the same biological sample were not treated as independent observations. Tukey’s test controlled for multiple pairwise comparisons within each individual endpoint. No global multiplicity correction was applied across the different behavioral and molecular endpoints. P values are reported using the following significance thresholds: *P* < 0.05, *P* < 0.01, *P* < 0.001, and *P* < 0.0001.

## Results

### MO Ameliorates Behavioral Deficits Associated with Aging

In the open-field test, OC mice spent significantly less time in the center zone than YC mice, whereas MO supplementation significantly increased center-zone exploration in aged mice (Fig. 1A and 1B).

Consistent with these age-associated behavioral alterations, OC mice also exhibited significantly lower spontaneous alternation in the Y-maze test than YC mice, indicating impaired spatial working memory-like performance (Fig. 1C and 1D). MO supplementation markedly improved spontaneous alternation in aged mice, bringing performance closer to the level observed in YC mice.

### MO Modulates Blood–Brain Barrier (BBB)-Associated Tight Junction Protein Expression in the Hippocampus

The expression of tight junction-associated proteins was evaluated to assess age-associated changes in BBB-associated protein expression. OC mice showed a pronounced reduction in the levels of BBB-associated tight junction proteins, including claudin-1, zonula occludens-1 (ZO-1), and occludin, compared with those in YC mice (Fig. 2A and 2B). In contrast, dietary MO supplementation was associated with higher expression of these tight junction proteins in aged mice. These findings indicate changes in BBB-associated tight junction protein expression but do not directly demonstrate functional BBB integrity or permeability.

Evaluation of senescence-associated markers in the hippocampus revealed a marked upregulation of p53, p21, p16, and plasminogen activator inhibitor-1 (PAI-1) in OC mice compared with YC mice (Fig. 2C and 2D). In contrast, dietary supplementation with MO significantly reduced the expression levels of these senescence markers in aged mice.

### MO Attenuates Age-Associated Neuroinflammatory Responses in the Hippocampus

Evaluation of inflammatory marker expression in the hippocampus showed pronounced upregulation of adhesion molecules, including intercellular adhesion molecule 1 (ICAM-1) and vascular cell adhesion molecule 1 (VCAM-1), along with increased levels of proinflammatory cytokines, including interleukin (IL)-1β, IL-6, and tumor necrosis factor-alpha (TNF-α) in OC mice compared with those in YC mice. In contrast, dietary MO supplementation significantly suppressed the expression of these inflammatory markers in OC mice (Fig. 3).

### MO Alleviates Age-Associated Oxidative Stress in the Hippocampus

Next, we assessed the expression of oxidative stress-related and antioxidant markers in the hippocampus (Fig. 4A and 4B). Compared with YC mice, OC mice exhibited a marked increase in the levels of pro-oxidant factors, including NADPH oxidase 4 (NOX4) and spermidine/spermine N1-acetyltransferase 1 (SAT1), accompanied by a significant reduction in the antioxidant enzyme superoxide dismutase 1 (SOD1). In contrast, dietary MO supplementation significantly suppressed NOX4 and SAT1 expression while increasing SOD1 levels in OC mice.

### MO Modulates Ferroptosis-Related Marker Expression in the Hippocampus of Aged Mice

To investigate whether MO modulates ferroptosis-associated changes during aging, key ferroptosis-associated markers were examined in the hippocampus. Compared with YC mice, OC mice exhibited a pronounced reduction in the expression of ferroptosis-protective proteins, glutathione peroxidase 4 (GPX4), SLC7A11 (xCT), and apoptosis-inducing factor 2 (AIFM2), a key regulator of ferroptosis resistance, accompanied by increased levels of acyl-CoA synthetase long-chain family member 1 (FACL2) in the hippocampus (Fig. 5A and 5B). In addition, lipid peroxidation was markedly elevated in OC mice, as evidenced by increased 4-HNE levels. Immunofluorescence staining further demonstrated increased 4-HNE and PTGS2 signals in the hippocampal region of OC mice compared with those in YC mice, consistent with increased lipid peroxidation and changes in ferroptosis-related markers during aging (Fig. 5C-5E). In contrast, dietary MO supplementation significantly increased GPX4, xCT, and AIFM2 expression while suppressing FACL2 levels in OC mice. Moreover, MO treatment was associated with reduced levels of lipid peroxidation-related markers, as demonstrated by decreased 4-HNE accumulation and reduced PTGS2 expression in both Western blotting and immunofluorescence analyses.

### MO Modulates Age-Associated Changes in Iron Metabolism-Related Protein Expression in the Hippocampus

We next investigated whether MO modulates age-associated alterations in iron homeostasis (Fig. 6A and 6B). OC mice showed pronounced dysregulation of iron metabolism-related protein expression in the hippocampus, including increased expression of transferrin receptor (TFR) and nuclear receptor coactivator 4 (NCOA4) and decreased expression of ferritin heavy chain (FtH), accompanied by reduced expression of heme oxygenase-1 (HO-1) and nuclear factor erythroid 2-related factor 2 (NRF2). Dietary MO supplementation reduced TFR and NCOA4 expression and partially restored FtH, HO-1, and NRF2 expression in OC mice.

### MO Modulates Ferritinophagy-Related Signaling in the Hippocampus of Aged Mice

To further elucidate the mechanisms underlying iron accumulation and ferroptosis-associated changes during aging, ferritinophagy-related signaling was examined in the hippocampus (Fig. 7A and 7B). Compared with the YC group, the OC group exhibited significant increases in ferritinophagy-associated markers, including Beclin1 and transcription factor EB (TFEB), along with a marked reduction in Krüppel-like factor 4 (KLF4). In contrast, dietary MO supplementation significantly suppressed the expression of Beclin1 and TFEB while restoring KLF4 levels in OC mice.

### MO Attenuates Age-Associated Apoptotic Signaling in the Hippocampus

Next, we investigated whether age-related activation of ferroptosis-associated and ferritinophagy-related pathways is accompanied by apoptotic cell death and apoptosis-associated signaling in the hippocampus (Fig. 8A and 8B). Compared with YC mice, OC mice exhibited a pronounced increase in pro-apoptotic markers, including B-cell lymphoma 2 (Bcl-2) associated X protein (BAX), cleaved caspase 3, cleaved caspase 7, and cleaved poly(ADP-ribose) polymerase (c-PARP), along with a concomitant reduction in the anti-apoptotic protein Bcl-2. In contrast, dietary MO supplementation significantly suppressed the expression of the pro-apoptotic proteins while restoring Bcl-2 levels in OC mice.

### MO Preserves Cholinergic Signaling and Synaptic Protein Expression in the Hippocampus of Aged Mice

To evaluate whether MO-mediated attenuation of cellular damage is associated with functional preservation, cholinergic-related markers and synaptic proteins were examined in the hippocampus (Fig. 9A and 9B). Compared with YC mice, OC mice exhibited a significant reduction in cholinergic markers, including choline acetyltransferase (ChAT) and acetylcholine receptor α3 (AChR-α3), along with increased acetylcholinesterase (AChE) expression, consistent with impaired cholinergic neurotransmission during aging. In addition, the expression of synaptic proteins synaptophysin (SYP) and postsynaptic density protein 95 (PSD-95) was markedly reduced in OC mice compared with that in YC mice. In contrast, dietary MO supplementation significantly restored ChAT and AChR-α3 levels while suppressing AChE expression in OC mice. Moreover, MO treatment effectively recovered the expression of SYP and PSD-95 in the aged hippocampus.

### MO Modulates Selected Alzheimer’s Disease-Related Protein Markers in the Hippocampus of Aged Mice

To examine selected Alzheimer’s disease (AD)-associated protein markers in the aged hippocampus, β-amyloid-related immunoreactivity and p-Tau (Ser404) levels were assessed by Western blotting (Fig. 10A and 10B). Compared with YC mice, OC mice exhibited increased β-amyloid-related immunoreactivity and p-Tau (Ser404) levels. In contrast, dietary MO supplementation was associated with significantly reduced β-amyloid-related immunoreactivity and p-Tau (Ser404) levels in OC mice.

## Discussion

Aging is associated with progressive hippocampal dysfunction characterized by oxidative stress, lipid peroxidation, iron dysregulation, and chronic neuroinflammation, which may increase vulnerability to neurodegenerative processes [8]. The hippocampus is particularly susceptible to age-associated metabolic imbalance because of its high oxygen consumption and limited antioxidant capacity [14]. During aging, reduced activity of endogenous antioxidant systems, together with excessive iron accumulation, may promote lipid peroxidation and disrupt redox homeostasis [15]. In this context, experimental aging models have shown that impairment of ferroptosis-protective systems and increased iron-dependent oxidative stress are associated with hippocampal neuronal injury and cognitive dysfunction [16]. In parallel, aging-associated cellular senescence and chronic neuroinflammation may further exacerbate oxidative stress and lipid peroxidation, creating a pathological microenvironment that favors ferroptosis [17]. These findings suggest that modulation of oxidative stress- and iron metabolism-associated pathways may contribute to the protective effects of MO against age-associated hippocampal dysfunction. Natural materials with antioxidant and anti-inflammatory properties may therefore have therapeutic potential for modulating these converging mechanisms [18]. In this context, the present study investigated whether dietary MO could attenuate hippocampal aging in association with changes in iron metabolism- and ferroptosis-related pathways.

Aging-related hippocampal dysfunction is influenced by the convergence of multiple pathological processes that reinforce one another over time [19]. In the aged hippocampus, cellular senescence and chronic neuroinflammation are associated with sustained inflammatory signaling and oxidative stress, which may contribute to increased neuronal vulnerability during aging [20]. Senescence-associated signaling is particularly important in this context because it is not merely a marker of aging but is also linked to sustained inflammatory activation and oxidative burden through the senescence-associated secretory phenotype and cytokine-mediated signaling pathways [21]. In parallel, inflammatory adhesion molecules may promote vascular–immune interactions and local tissue stress, further contributing to redox imbalance [22]. In addition, disruption of BBB-associated tight junction proteins has been linked to neuroinflammatory alterations and hippocampal dysfunction during aging [23]. In the present study, MO supplementation was associated with reduced expression of senescence-associated and inflammatory markers and partial restoration of BBB-associated tight junction protein expression in the hippocampus (Fig. 2). These concurrent changes suggest that MO supplementation was associated with modulation of senescence-, inflammation-, and tight junction-related protein expression in the aged hippocampus. However, the present data do not establish functional preservation of the BBB or a causal relationship between tight junction protein expression and the other molecular effects of MO.

Redox imbalance and dysregulation of iron metabolism have been increasingly associated with oxidative stress and susceptibility to ferroptosis in the aging hippocampus [15]. During aging, impairment of endogenous antioxidant defense systems may shift the cellular redox balance toward a more pro-oxidant state, thereby enhancing lipid peroxidation and increasing susceptibility to oxidative damage [24]. In addition, age-associated alterations in iron handling may contribute to oxidative stress through iron-dependent oxidative reactions and lipid peroxidation [25]. Experimental studies using aging models and aged mice have reported that hippocampal oxidative stress, altered iron handling, and changes in ferroptosis-related proteins are associated with cognitive impairment [16, 26]. In the present study, aging was associated with altered expression of oxidative stress-related and iron metabolism-related proteins, accompanied by increased expression of lipid peroxidation markers in the hippocampus (Figs. 4 and 6). MO supplementation modulated these alterations and was associated with restoration of ferroptosis-associated regulatory protein expression, suggesting effects that may extend beyond general antioxidant activity. Furthermore, MO supplementation was associated with reduced lipid peroxidation and modulation of iron metabolism-related pathways in the aged hippocampus (Figs. 5 and 6). Collectively, these findings suggest that modulation of oxidative stress- and iron metabolism-associated pathways may contribute to the effects of MO on age-associated hippocampal dysfunction. These coordinated changes suggest an association between the effects of MO and modulation of oxidative stress- and iron metabolism-related processes; however, they do not demonstrate that these pathways causally mediate the effects of MO.

Ferroptosis has been increasingly associated with aging-related iron dysregulation, lipid peroxidation, and neuronal vulnerability in the hippocampus [9]. In the aging hippocampus, sustained oxidative stress and altered iron metabolism may impair ferroptosis-protective systems, thereby contributing to increased lipid peroxidation and ferroptosis-associated molecular alterations [10]. Although direct evidence linking ferritinophagy to natural hippocampal aging remains limited, NCOA4-mediated ferritinophagy has been shown to promote ferritin degradation, intracellular iron release, and ferroptosis-related neuronal injury in experimental hippocampal models [27]. In the present study, aging was associated with coordinated alterations in ferritinophagy-associated proteins and ferroptosis-related markers, accompanied by increased lipid peroxidation- and apoptosis-related marker expression. Dietary MO supplementation was associated with partial normalization of these marker changes (Figs. 5–8). Because ferritinophagic flux, intracellular labile iron, and ferroptotic cell death were not directly measured, these findings should be interpreted as associations among ferritinophagy-, iron metabolism-, ferroptosis-, and apoptosis-related markers rather than as evidence of a causal ferroptosis–ferritinophagy–apoptosis continuum.

Preservation of neuronal function is closely associated with maintenance of hippocampus-dependent cognitive processes during aging [17]. The cholinergic system plays a central role in learning and memory, and aging-associated alterations in cholinergic signaling have been linked to cognitive decline [28]. In addition, oxidative stress and lipid peroxidation may contribute to impairment of cholinergic neurotransmission and synaptic protein expression in the aged hippocampus [8]. Accordingly, alterations in cholinergic markers and synaptic proteins may reflect not only molecular changes associated with aging but also the functional consequences of cumulative neuronal stress [29]. In the present study, MO supplementation was associated with changes in ferroptosis-, ferritinophagy-, and apoptosis-related markers, together with preservation of cholinergic and synaptic protein expression. Although these concurrent changes may be biologically related, the present experimental design does not establish that modulation of ferroptosis or ferritinophagy directly caused the changes in cholinergic and synaptic proteins (Figs. 8 and 9). Preservation of synaptic integrity may therefore be associated with the maintenance of cholinergic system components observed in MO-supplemented aged mice (Fig. 9). Because cholinergic neurotransmission and synaptic integrity are central to hippocampus-dependent cognitive processes, the observed preservation of cholinergic and synaptic protein expression may be associated with the improved cognitive-related behavioral performance [30]. Consistent with this interpretation, maintenance of cholinergic signaling and synaptic protein expression may be associated with the improved hippocampus-dependent behavioral performance observed following MO supplementation (Fig. 1). These findings suggest that modulation of iron- and ferroptosis-associated processes may be related to the preservation of molecular markers of cholinergic and synaptic function, supporting the functional relevance of ferroptosis-associated pathways in hippocampal aging.

In addition to functional impairment, aging is associated with the accumulation of neurodegeneration-related pathological proteins, including amyloid-β and phosphorylated tau, which have been linked to oxidative stress and iron dysregulation [31]. Previous studies have suggested that lipid peroxidation and iron-dependent oxidative stress may contribute to amyloidogenic processing and tau-related pathology, thereby linking ferroptotic stress to broader neurodegenerative changes [10]. In the present study, MO supplementation was associated with reduced β-amyloid-related immunoreactivity and p-Tau (Ser404) levels in the aged hippocampus (Fig. 10). Because the present study used naturally aged mice rather than an AD model and did not include histological assessment of amyloid plaques, tau aggregation, or neuronal loss, these findings should be interpreted only as changes in selected AD-related protein markers. They do not demonstrate attenuation of amyloid plaque burden, tau pathology, AD-like pathology, or neurodegenerative progression.

Taken together, these findings suggest that modulation of ferroptosis-associated and iron metabolism-related pathways may be associated with preservation of hippocampal function and reduced expression of selected neurodegeneration-related protein markers during aging. These observations support the potential relevance of oxidative stress- and ferroptosis-associated mechanisms in age-associated hippocampal dysfunction.

Several limitations of the present study should be considered. First, the three-group experimental design did not include a ferroptosis inhibitor, iron chelator, or other positive comparator to establish the causal involvement of ferroptosis or iron dysregulation. Second, direct measurements of total or labile iron, lipid ROS, glutathione levels, GPX activity, and ferroptotic cell death were not performed. The present findings are therefore limited to treatment-associated changes in iron metabolism- and ferroptosis-related markers and do not demonstrate direct inhibition of ferroptosis. Further studies incorporating direct biochemical measurements and pharmacological or genetic interventions are needed to determine whether changes in iron metabolism or ferroptosis causally contribute to the effects of MO in the aged hippocampus. Third, only male mice were included in the present study. Therefore, the findings cannot be generalized to female mice, and potential sex-specific differences in age-associated hippocampal alterations or responses to MO could not assessed. Future studies including both male and female mice are needed to determine whether the effects of MO differ by sex. An additional limitation is that the behavioral assessment was limited to Y-maze spontaneous alternation and open-field center-zone activity. Although these tests provide information on working memory-like performance and exploratory/anxiety-like behavior, additional hippocampus-dependent behavioral tasks would strengthen the functional interpretation of the present findings. Future studies incorporating spatial learning and recognition paradigms are warranted. Additionally, formal dose-response and toxicological evaluations were not performed, warranting further studies to determine the optimal dose and safety profile of MO.

## Conclusion

The present study found that dietary MO was associated with attenuation of age-associated hippocampal dysfunction and modulation of oxidative stress-, iron metabolism-, ferroptosis-, and apoptosis-related markers in aged mice. MO supplementation was also associated with preservation of cholinergic and synaptic protein expression and with lower β-amyloid-related immunoreactivity and p-Tau (Ser404) levels in the aged hippocampus. Collectively, these findingssuggest that the effects of MO on age-associated hippocampal alterations were accompanied by coordinated changes in oxidative stress-, iron metabolism-, and ferroptosis-related markers. However, the present data do not establish iron dysregulation or ferroptosis as causal mediators of the observed effects. Further studies are warranted to determine the translational relevance of these findings and the mechanisms underlying the effects of MO in aging and neurodegenerative conditions.

## Supplemental Materials

Supplementary data for this paper are available on-line only at http://jmb.or.kr.



## Figures and Tables

**Fig. 1:**
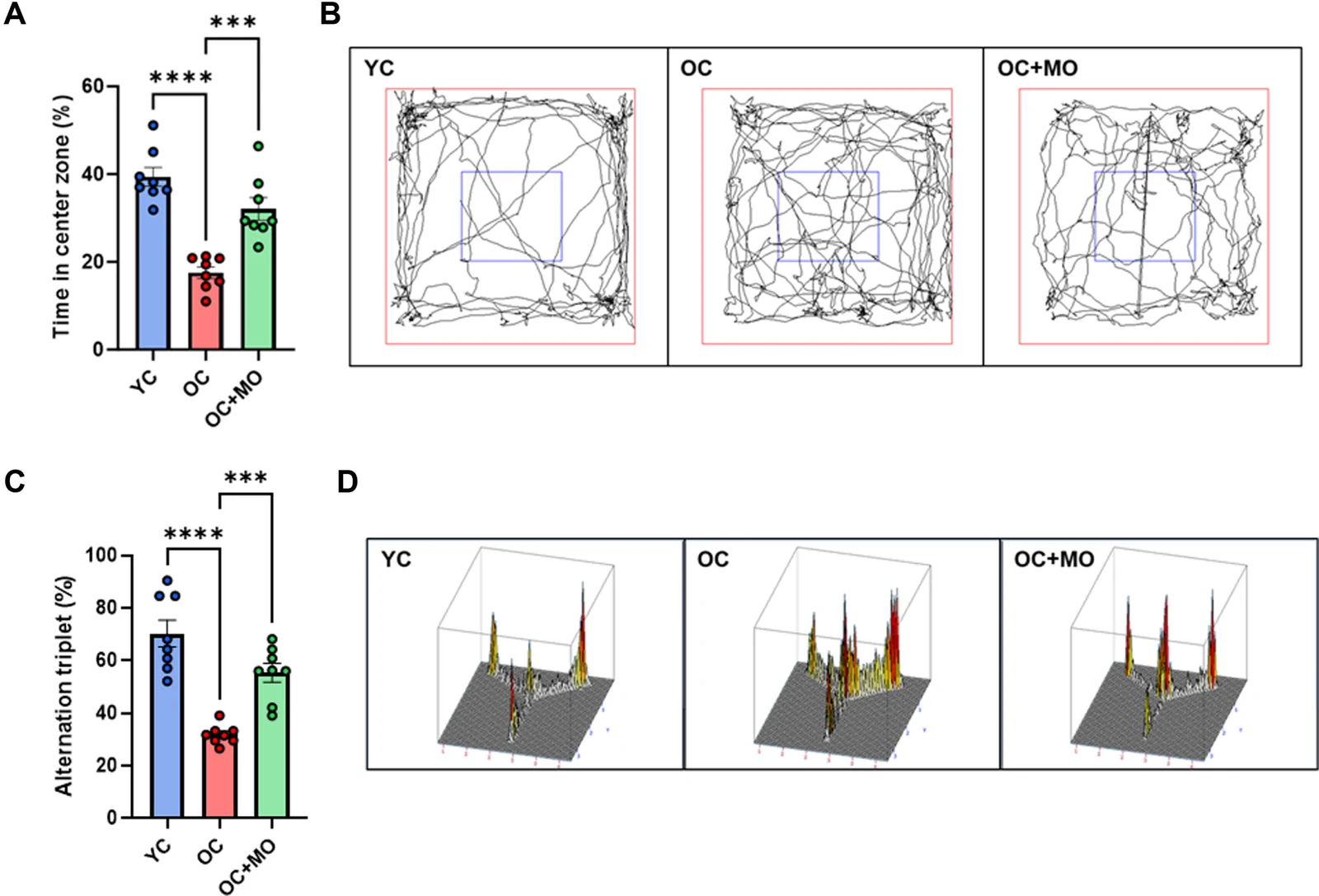
*Magnolia kobus* DC. (MO) ameliorates age-associated behavioral deficits in mice. (**A**) Time spent in the central zone during the open-field test. (**B**) Representative tracking images from the open-field test. (**C**) Spontaneous alternation performance (%) in the Y-maze test. (**D**) Representative activity maps from the Y-maze test. Data are expressed as mean ± SD (n = 8 per group). Statistical significance was determined by one-way ANOVA followed by Tukey’s multiple comparisons test. ***P < 0.001, ****P < 0.0001 vs. indicated groups.

**Fig. 2:**
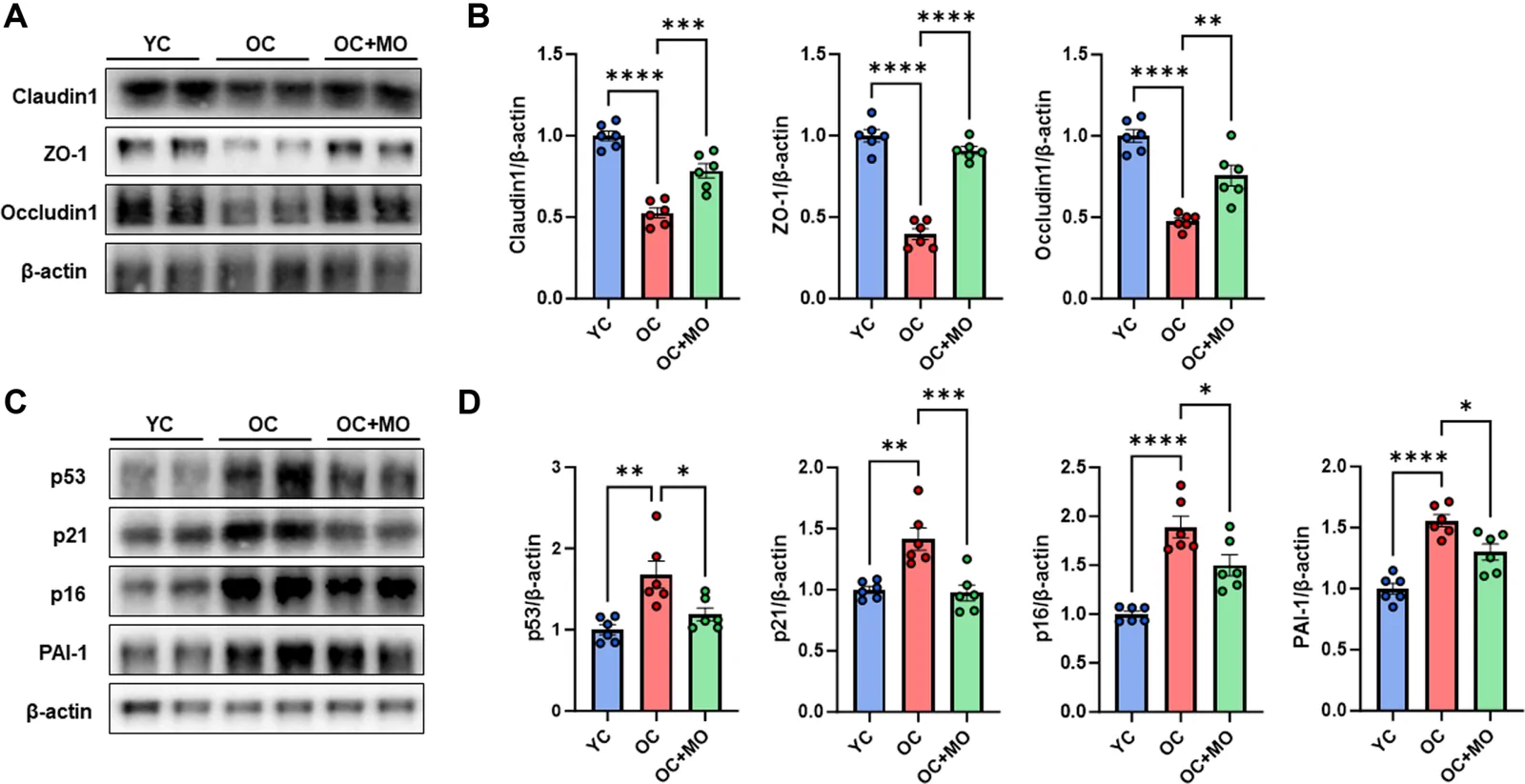
*Magnolia kobus* DC. (MO) modulates blood–brain barrier (BBB)- and senescence-associated protein expression in the hippocampus of aged mice. (**A**) Representative Western blot images of tight junction-associated proteins, including claudin-1, zonula occludens-1 (ZO-1), and occludin-1. (**B**) Relative protein expression normalized to β-actin. (**C**) Representative Western blot images of senescence-associated markers, including p53, p21, p16, and plasminogen activator inhibitor-1 (PAI-1). (**D**) Relative protein expression normalized to β-actin. Data are expressed as mean ± SD (n = 6 per group). Statistical significance was determined by one-way ANOVA followed by Tukey’s multiple comparisons test. *P < 0.05, **P < 0.01, ***P < 0.001, ****P < 0.0001 vs. indicated groups.

**Fig. 3:**
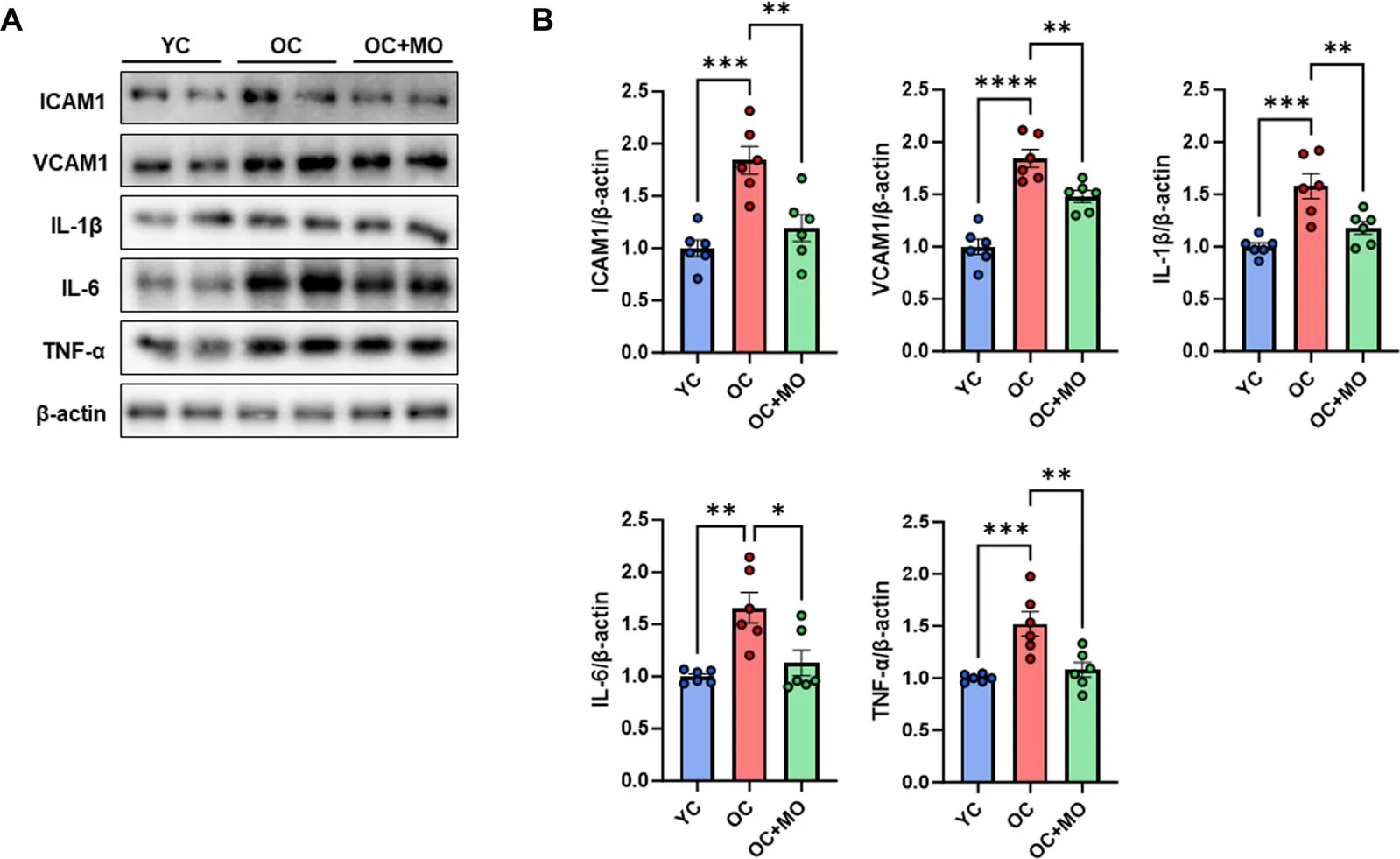
*Magnolia kobus* DC. (MO) suppresses age-associated neuroinflammatory responses in the hippocampus of aged mice. (**A**) Representative Western blot images of inflammatory markers, including intercellular adhesion molecule 1 (ICAM-1), vascular cell adhesion molecule 1 (VCAM-1), interleukin (IL)-1β, IL-6, and tumor necrosis factor-α (TNF-α). (**B**) Relative protein expression normalized to β-actin. Data are expressed as mean ± SD (n = 6 per group). Statistical significance was determined by one-way ANOVA followed by Tukey’s multiple comparisons test. *P < 0.05, **P < 0.01, ***P < 0.001, ****P < 0.0001 vs. indicated groups.

**Fig. 4:**
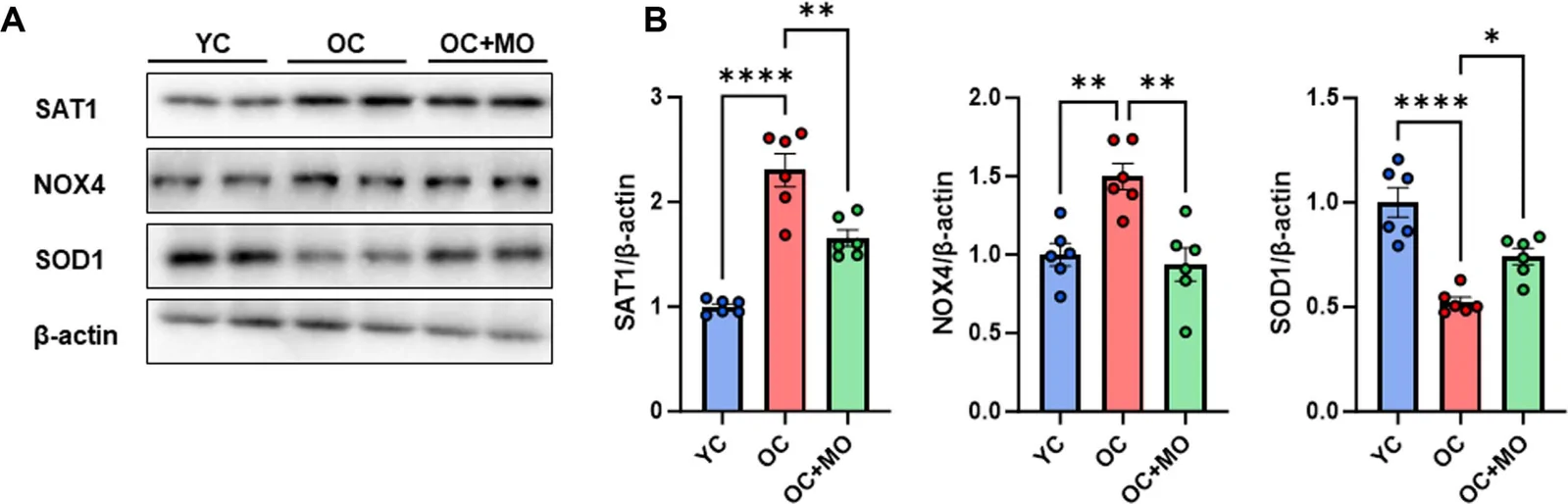
*Magnolia kobus* DC. (MO) attenuates age-associated oxidative stress-related signaling in the hippocampus of aged mice. (**A**) Representative Western blot images of oxidative stress-related proteins, including NADPH oxidase 4 (NOX4), spermidine/spermine N1-acetyltransferase 1 (SAT1), and superoxide dismutase 1 (SOD1). (**B**) Relative protein expression normalized to β-actin. Data are expressed as mean ± SD (n = 6 per group). Statistical significance was determined by one-way ANOVA followed by Tukey’s multiple comparisons test. *P < 0.05, **P < 0.01, ***P < 0.001, ****P < 0.0001 vs. indicated groups.

**Fig. 5:**
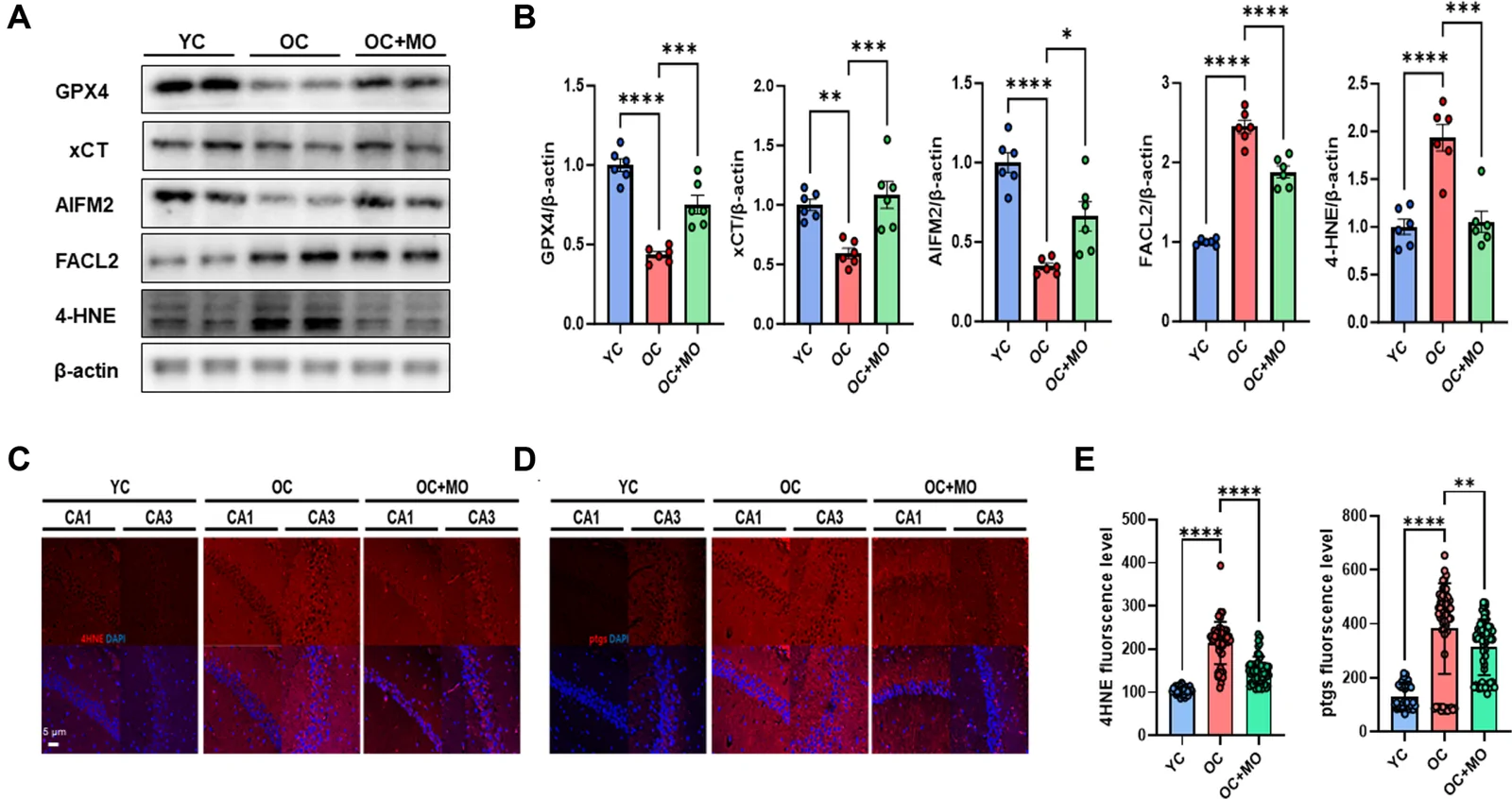
*Magnolia kobus* DC. (MO) modulates ferroptosis-related marker expression in the hippocampus of aged mice. (**A**) Representative Western blot images of ferroptosis-related proteins, including glutathione peroxidase 4 (GPX4), SLC7A11 (xCT), apoptosis-inducing factor 2 (AIFM2), acyl-CoA synthetase long-chain family member 1 (FACL2), and 4-hydroxynonenal (4-HNE). (**B**) Relative protein expression normalized to β-actin. (**C**) Representative immunofluorescence images of 4-HNE in the hippocampus. (**D**) Representative immunofluorescence images of prostaglandin-endoperoxide synthase 2 (PTGS2) in the hippocampus. (**E**) Representative immunofluorescence images of 4-HNE and PTGS2 and in the hippocampus. For fluorescence quantification, more than 30 randomly selected fields of view from two independent biological samples were analyzed per group (>30 fields/group). Statistical significance was determined by one-way ANOVA followed by Tukey’s multiple comparisons test. **P < 0.01, ****P < 0.0001 vs. indicated groups.

**Fig. 6:**
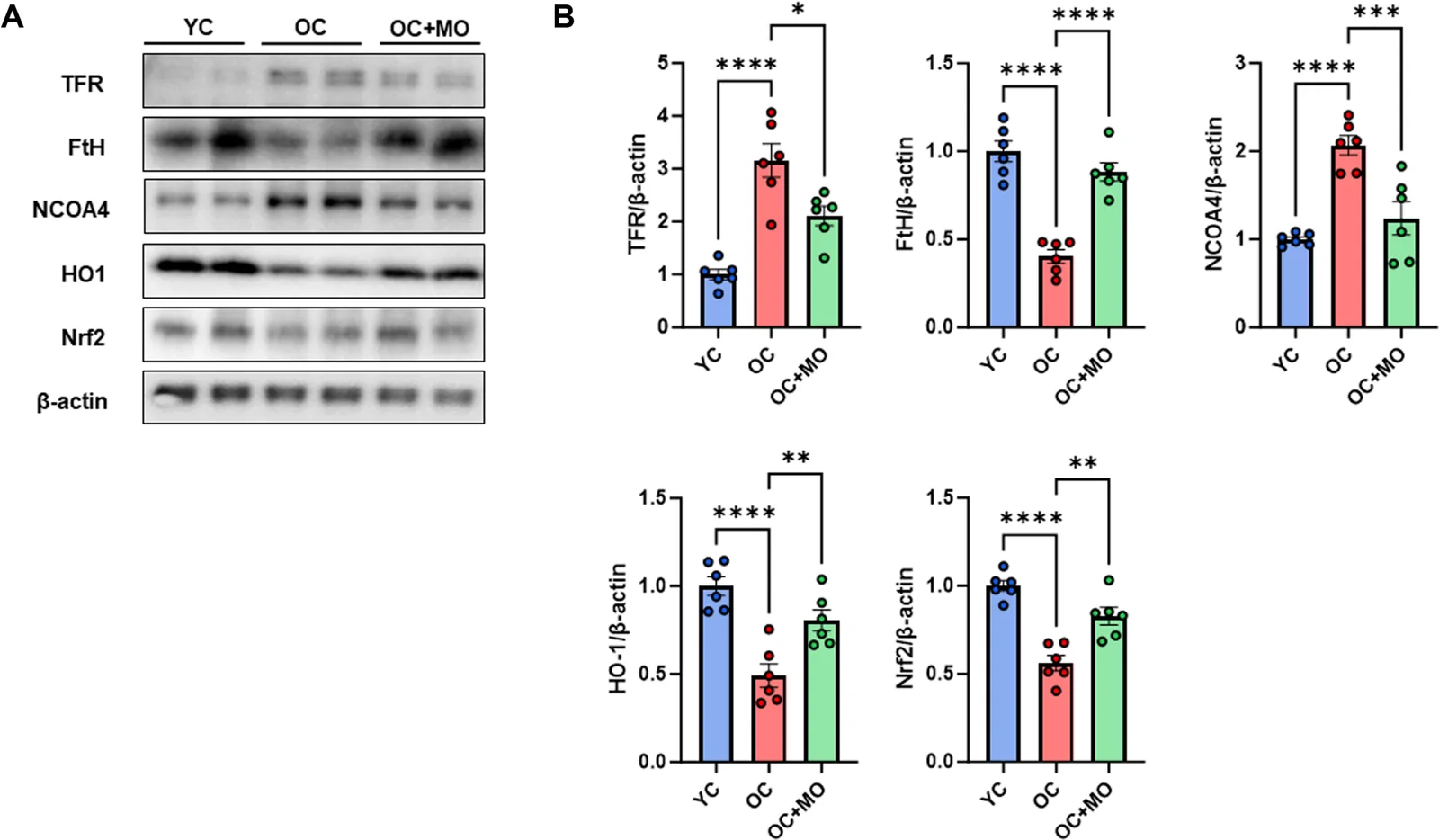
*Magnolia kobus* DC. (MO) modulates age-associated changes in iron metabolism-related protein expression in the hippocampus of aged mice. (**A**) Representative Western blot images of iron metabolism-related proteins, including transferrin receptor (TFR), ferritin heavy chain (FtH), nuclear receptor coactivator 4 (NCOA4), heme oxygenase-1 (HO-1), and nuclear factor erythroid 2-related factor 2 (NRF2). (**B**) Relative protein expression normalized to β-actin. Data are expressed as mean ± SD (n = 6 per group). Statistical significance was determined by one-way ANOVA followed by Tukey’s multiple comparisons test. *P < 0.05, **P < 0.01, ***P < 0.001, ****P < 0.0001 vs. indicated groups.

**Fig. 7:**
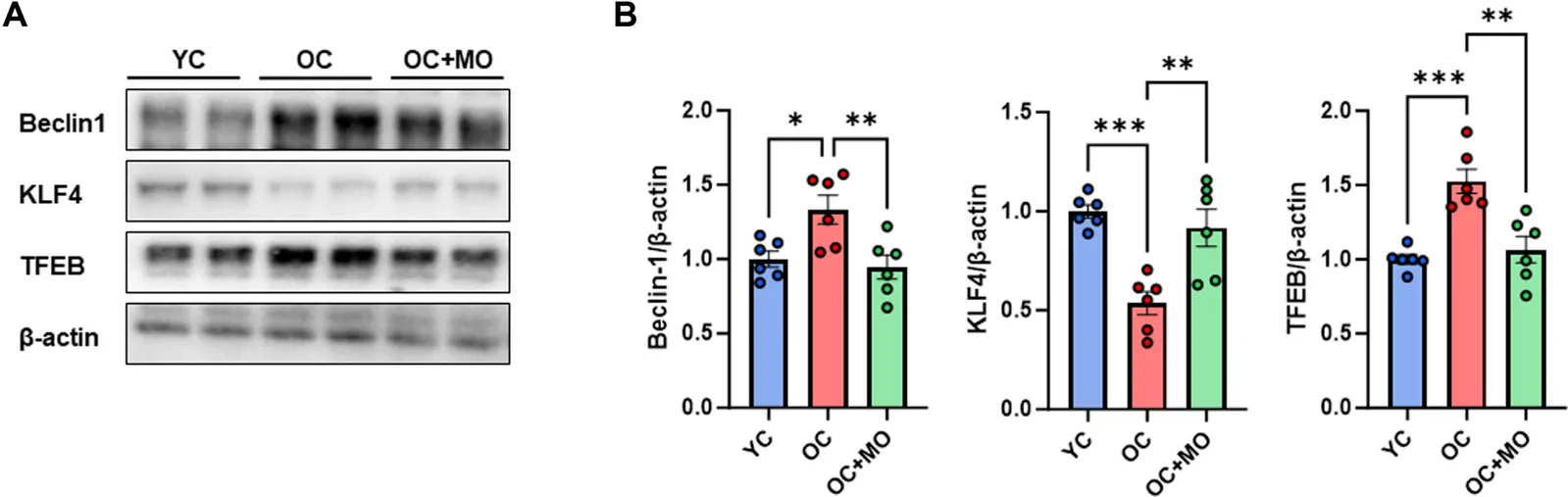
*Magnolia kobus* DC. (MO) modulates ferritinophagy-associated signaling in the hippocampus of aged mice. (**A**) Representative Western blot images of ferritinophagy-associated proteins, including Beclin1, Krüppel-like factor 4 (KLF4), and transcription factor EB (TFEB). (**B**) Relative protein expression normalized to β-actin. Data are expressed as mean ± SD (n = 6 per group). Statistical significance was determined by one-way ANOVA followed by Tukey’s multiple comparisons test. *P < 0.05, **P < 0.01, ***P < 0.001, ****P < 0.0001 vs. indicated groups.

**Fig. 8:**
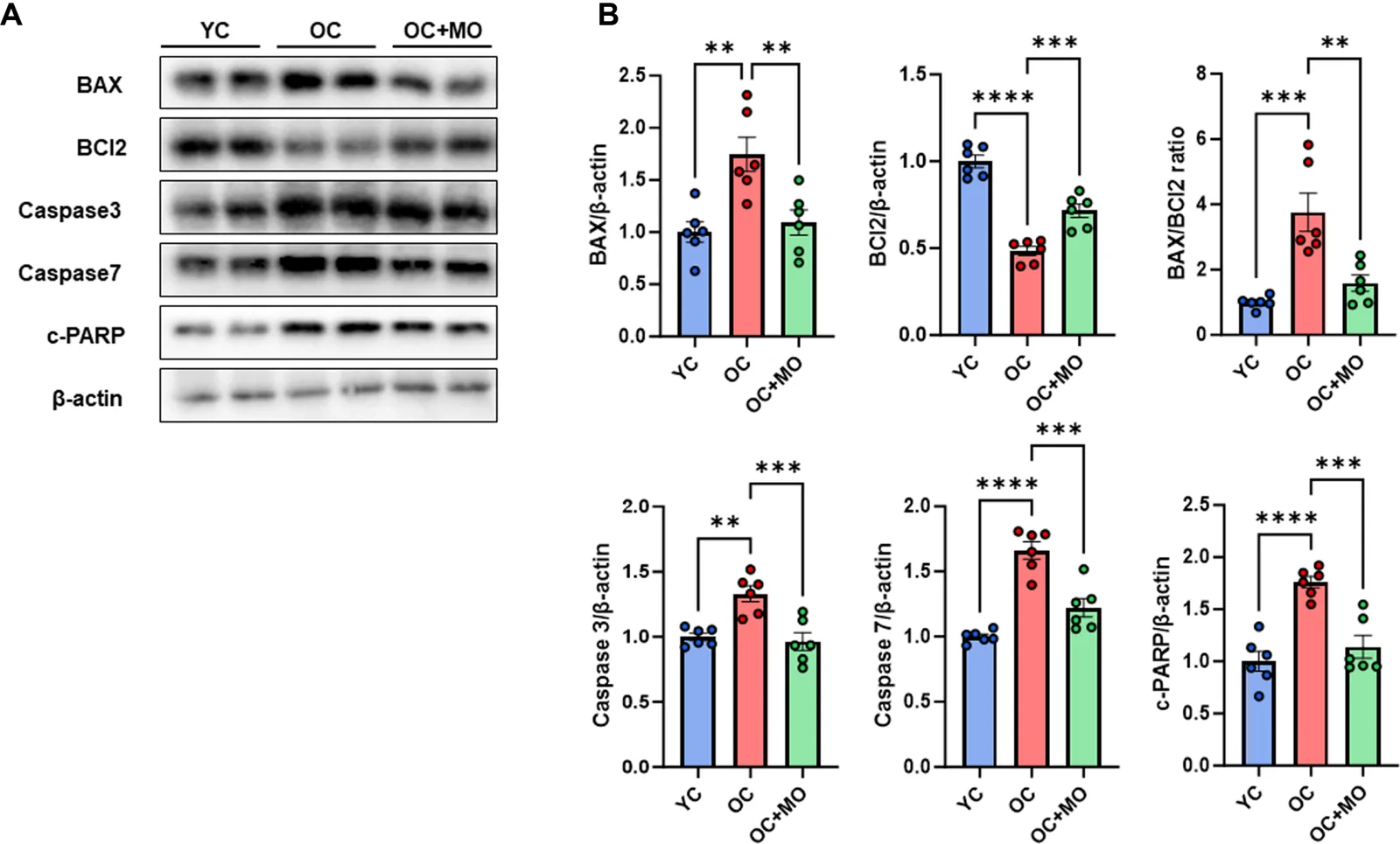
*Magnolia kobus* DC. (MO) attenuates age-associated apoptotic signaling in the hippocampus of aged mice. (**A**) Representative Western blot images of apoptosis-related proteins, including B-cell lymphoma 2 (Bcl-2)-associated X protein (BAX), Bcl-2, cleaved caspase 3, cleaved caspase 7, and cleaved poly(ADP-ribose) polymerase (c-PARP). (**B**) Relative protein expression normalized to β-actin. Data are expressed as mean ± SD (n = 6 per group). Statistical significance was determined by one-way ANOVA followed by Tukey’s multiple comparisons test. *P < 0.05, **P < 0.01, ***P < 0.001, ****P < 0.0001 vs. indicated groups.

**Fig. 9:**
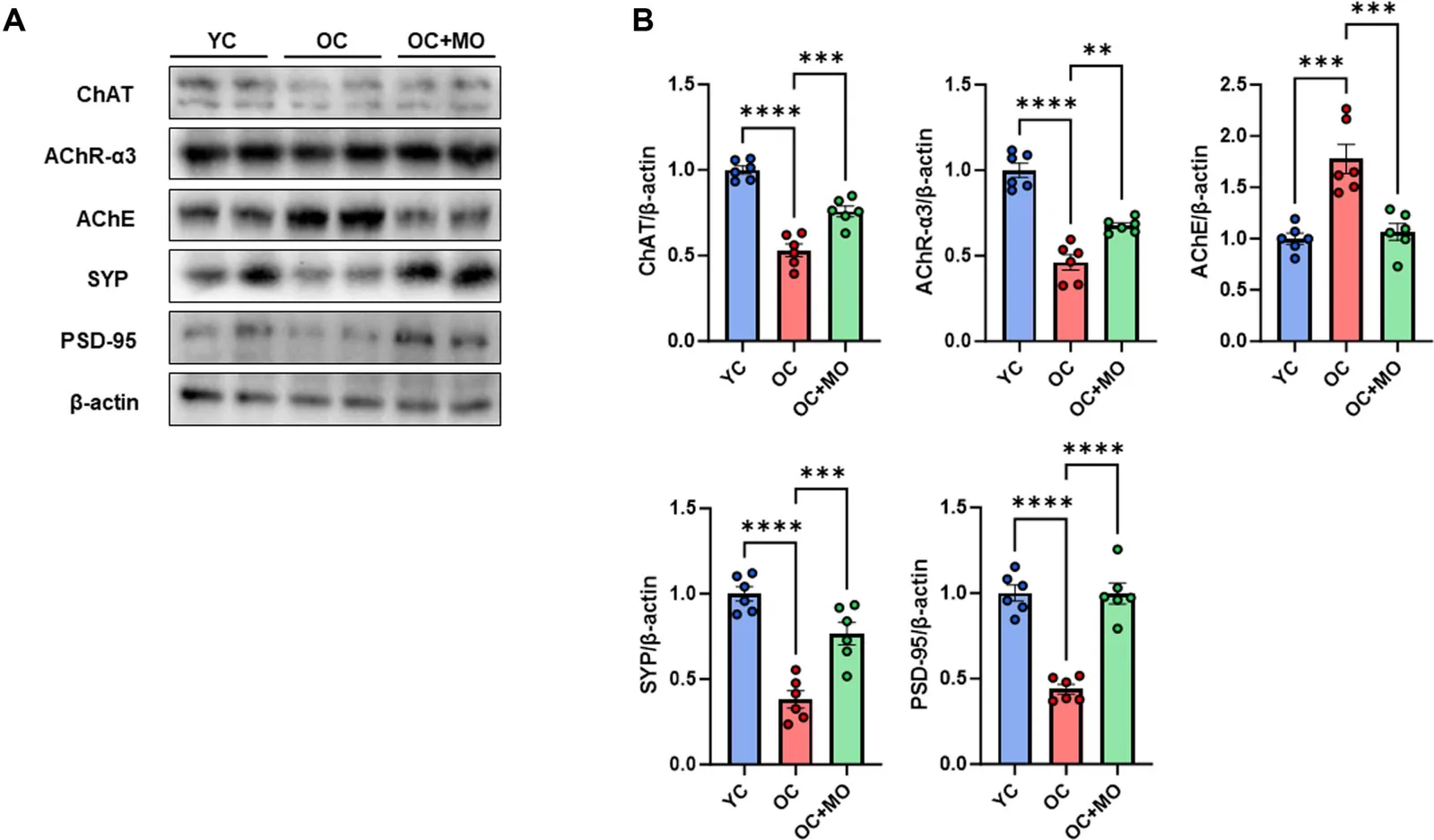
*Magnolia kobus* DC. (MO) preserves cholinergic signaling and synaptic integrity in the hippocampus of aged mice. (**A**) Representative Western blot images of cholinergic and synaptic markers, including choline acetyltransferase (ChAT), acetylcholine receptor α3 (AChR-α3), acetylcholinesterase (AChE), synaptophysin (SYP), and postsynaptic density protein 95 (PSD-95). (**B**) Relative protein expression normalized to β-actin. Data are expressed as mean ± SD (n = 6 per group). Statistical significance was determined by one-way ANOVA followed by Tukey’s multiple comparisons test. *P < 0.05, **P < 0.01, ***P < 0.001, ****P < 0.0001 vs. indicated groups.

**Fig. 10:**
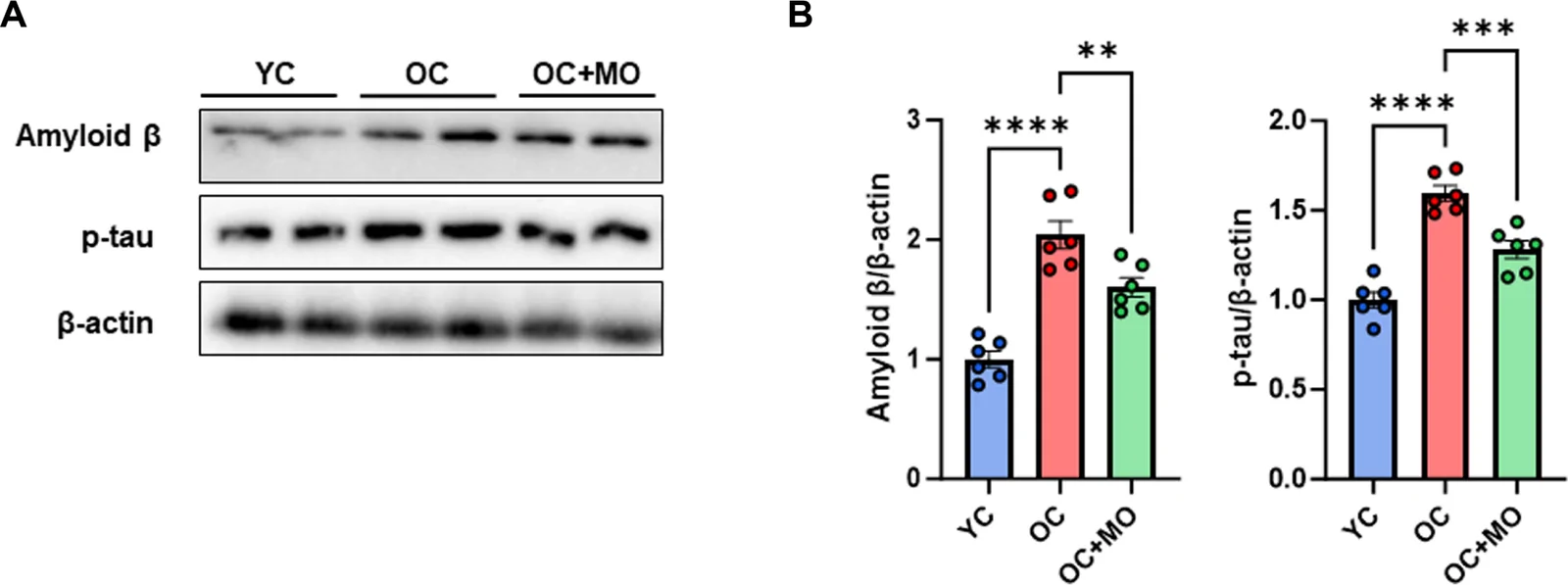
*Magnolia kobus* DC. (MO) reduces age-associated accumulation of Alzheimer’s disease-related markers in the hippocampus of aged mice. (**A**) Representative Western blot images of β-amyloid-related immunoreactivity and phosphorylated tau (Ser404). (**B**) Relative protein expression normalized to β-actin. Data are expressed as mean ± SD (n = 6 per group). Statistical significance was determined by one-way ANOVA followed by Tukey’s multiple comparisons test. *P < 0.05, **P < 0.01, ***P < 0.001, ****P < 0.0001 vs. indicated groups.
