# Supplementary material for: Dietary Magnolia kobus DC. Attenuates Age-Associated Hippocampal Alterations in Mice in Association with Modulation of Iron Metabolism- and Ferroptosis-Related Markers
Source: J Microbiol Biotechnol. 2026 Sep 15;36:e2606032. doi: 10.4014/jmb.2606.06032 (PMC13630537; doi:10.4014/jmb.2606.06032)
Supplement: Supplementary file 1 [file jmb-36-e2606032-supple.pdf]

J. Microbiol. Biotechnol. 2026. 36: e2606032

https://doi.org/10.4014/jmb.2606.06032

Supplemental data S1. List of antibody details used in this study.

Antibody	Catalog	Molecular Weight (kDa)	Concentration	Manufacturer
NOX4	ab109225	67	1:1,000	Abcam (Cambridge, UK)
TFR	ab269513	84	1:1,000	Abcam
Nrf2	ab62352	68 (predicted); ~85–100 observed)	1:1,000	Abcam
GPX4	ab125066	22	1:1,000	Abcam
xCT	ab175186	55	1:1,000	Abcam
NCOA4	ab314553	70	1:1,000	Abcam
4-HNE	ab46545	N/A (multiple protein adducts)	1:1,000	Abcam
Ptgs2	ab15191	~69	1:1,000	Abcam
Beclin1	ab210498	~52	1:1,000	Abcam
KLF4	ab106629	~55	1:1,000	Abcam
Claudin1	sc-166338	22	1:1,000	Santa Cruz Biotechnology (Dallas, TX, USA)
ZO-1	sc-33725	~220	1:1,000	Santa Cruz Biotechnology
Occludin1	sc-133256	60-82	1:1,000	Santa Cruz Biotechnology
p53	sc-126	53	1:1,000	Santa Cruz Biotechnology
p16	sc-166760	16	1:1,000	Santa Cruz Biotechnology.
p21	sc-6246	21	1:1,000	Santa Cruz Biotechnology.
PAI-1	sc-5297	~47	1:1,000	Santa Cruz Biotechnology.
β-actin	sc-47778	43	1:1,000	Santa Cruz Biotechnology
ICAM1	sc-8439	~90	1:1,000	Santa Cruz Biotechnology
VCAM1	sc-13160	~110	1:1,000	Santa Cruz Biotechnology
IL-6	sc-130326	~21-28	1:1,000	Santa Cruz Biotechnology
TNF-α	sc-1350	~26	1:1,000	Santa Cruz Biotechnology
IL—1β	Sc-1250	31	1:1000	Santa Cruz Biotechnology
SOD-1	sc-17767	~16	1:1,000	Santa Cruz Biotechnology
FtH	sc-376594	~21	1:1,000	Santa Cruz Biotechnology
AIFM2	HPA042309	41	1:1000	Atlas Antibodies

BAX	sc-7480	23	1:1,000	Santa Cruz Biotechnology
BCl2	sc-509	~26	1:1,000	Santa Cruz Biotechnology.
Caspase7	sc-56063	~35	1:1,000	Santa Cruz Biotechnology.
c-PARP	sc-56196	~89	1:1,000	Santa Cruz Biotechnology
AChR-α3	sc-365479	~57	1:1,000	Santa Cruz Biotechnology
AChE	sc-373901	~68	1:1,000	Santa Cruz Biotechnology.
SYP	sc-55507	~38	1:1,000	Santa Cruz Biotechnology.
PSD-95	sc-71935	~95	1:1,000	Santa Cruz Biotechnology.
Amyloid β	sc-28365	4–46	1:1,000	Santa Cruz Biotechnology
p-tau	sc-12952	~55-80	1:1,000	Santa Cruz Biotechnology
HO-1	10701-1-ap	33	1:1,000	Proteintech, (San Diego, CA, USA)
FACL2	#4047	~79	1:1,000	Cell Signaling Technology (Danvers, MA, USA)
TFEB	#32361s	65-70	1:1,000	Cell Signaling Technology
SAT1	16992	~20	1:1,000	Thermo Fisher Scientific (Waltham, MA, USA)
Caspase3	CSB-PA05689A0Rb	~32	1:1,000	Cusabio (Hubei, China)
ChAT	20747-1AP	~70	1:1,000	Bioneer (Daejeon, Korea)
Secondary anti-rabbit	7074S		1:5,000	Cell Signaling Technology
Secondary anti-mouse	7076S		1:5,000	Cell Signaling Technology
Secondary anti-goat	sc-2020		1:5,000	Santa Cruz Biotechnology
